# Analytical Evaluation of a Point-of-Care Platform for Glycated Hemoglobin and N-Terminal Pro-B-Type Natriuretic Peptide Testing in Cardiometabolic Care

**DOI:** 10.14740/cr2220

**Published:** 2026-06-05

**Authors:** Perrin Ngougni Pokem, Ali Khatib, Iman Azariouh Kaddouri, Damien Gruson

**Affiliations:** a Department of Clinical Biochemistry, Cliniques Universitaires St-Luc and Universite Catholique de Louvain, Brussels, Belgium; b Pole de Recherche en Endocrinologie, Diabete et Nutrition, Institut de Recherche Experimentale et Clinique, Cliniques Universitaires St-Luc and Universite Catholique de Louvain, Brussels, Belgium; c UCL-SSH/IRIS-L/IRIB-Institut de Recherches Interdisciplinaires sur Bruxelles, Brussels, Belgium; d Institute Roger Lambion, Brussels, Belgium; e Department of Laboratory Medicine, Cliniques Universitaires Saint-Luc, UCLouvain, Brussels, Belgium; f These authors contribute equally to the article.

**Keywords:** Point-of-care testing, Biomarkers, Diabetes, Heart failure, HbA1c, NT-proBNP

## Abstract

**Background:**

Heart failure is a frequent complication of diabetes, highlighting the importance of integrated cardiometabolic assessment. Glycated hemoglobin (HbA1c) and N-terminal pro-B-type natriuretic peptide (NT-proBNP) represent complementary biomarkers reflecting glycemic control and myocardial stress. Point-of-care testing (POCT) platforms may facilitate decentralized diagnostics and shorten analytical turnaround time. The aim of this study was to evaluate the analytical performance of the AFIAS-3 point-of-care fluorescence immunoassay platform for HbA1c and NT-proBNP measurements in comparison with established central laboratory methods.

**Methods:**

This single-center verification study included 50 patient samples for each analyte. HbA1c measurements obtained using AFIAS-3 were compared with Tosoh G8 high-performance liquid chromatography (HPLC), while NT-proBNP measurements were compared with the cobas 8000 electrochemiluminescence immunoassay. Analytical precision and accuracy were assessed using manufacturer quality control materials. Method comparison was performed using Passing–Bablok regression, Bland–Altman analysis, and Spearman correlation. Agreement at clinical thresholds was evaluated using Cohen’s kappa statistics.

**Results:**

For HbA1c, analytical precision was excellent, with coefficients of variation of 1.9% and 1.8%. Correlation with the reference method was strong (r = 0.98), and the mean bias was minimal (−0.03%). Agreement at the diagnostic threshold of 6.5% was excellent (κ = 0.92). For NT-proBNP, coefficients of variation were 5.3% and 15.4%. Correlation with the central laboratory method was very high (r = 0.99), although proportional bias was observed at higher concentrations (slope 1.24). Agreement at clinically relevant thresholds remained substantial.

**Conclusions:**

The AFIAS-3 POCT platform demonstrated excellent analytical agreement for HbA1c and acceptable agreement for NT-proBNP at clinically relevant thresholds. Dual biomarker POCT may support decentralized cardiometabolic diagnostics, although NT-proBNP values at higher concentrations should be interpreted cautiously.

## Introduction

Diabetes is a major risk factor for the development of heart failure, increasing its incidence by two- to four-fold depending on sex and glycemic control [1, 2]. Chronic hyperglycemia contributes to myocardial structural and functional alterations, including interstitial fibrosis and diabetic cardiomyopathy [3, 4]. Early identification of cardiovascular complications in patients with diabetes therefore remains a major clinical priority.

Two biomarkers play important and complementary roles in cardiometabolic assessment. Glycated hemoglobin (HbA1c) represents the standard biomarker for evaluating long-term glycemic control and is widely used for both diagnosis and monitoring of diabetes [3]. N-terminal pro-B-type natriuretic peptide (NT-proBNP) reflects myocardial wall stress and is widely used in the diagnosis and risk stratification of heart failure [5, 6]. Current international guidelines recommend the use of natriuretic peptides to support diagnostic assessment and guide referral for cardiac imaging.

Recent advances in point-of-care testing (POCT) technologies have enabled the measurement of biomarkers directly at or near the patient, outside traditional centralized laboratory environments [7]. Such decentralized diagnostic tools may reduce analytical turnaround time and support diagnostic workflows in outpatient clinics, primary care, and emergency settings.

Before implementation in clinical practice, however, POCT platforms must demonstrate analytical performance comparable to established laboratory assays. The objective of the present study was therefore to verify the analytical performance of the AFIAS-3 POCT fluorescence immunoassay (FIA) platform for the measurement of HbA1c and NT-proBNP in comparison with established central laboratory methods.

## Materials and Methods

### Ethics

This single-center verification study was conducted at Cliniques Universitaires Saint-Luc (Brussels, Belgium). The protocol complied with the Declaration of Helsinki and applicable regulations. Approval was granted by the local ethics committee (Reference number: B4032024000084). Data were pseudonymized prior to analysis.

### Specimens

Two measurement windows were used: December 2024 for NT-proBNP and February 2025 for HbA1c. For the method comparison, samples were collected as follows: 1) HbA1c in venous whole blood collected in EDTA K3 tubes (Monovette EDTA K3E, 3.4 mL; Sarstedt, Germany); 2) NT-proBNP in lithium-heparin plasma (Monovette Li Heparin LH, 2.7 mL; Sarstedt, Germany). Routine pre-analytical processing was applied. Samples with clotting, hemolysis, lipemia, or insufficient volume were excluded.

### Reagents and instruments

POCT was performed on AFIAS-3 (Boditech Med, Chuncheon, Republic of Korea), a cartridge-based FIA platform (sample volume: 10 µL for HbA1c and 100 µL for NT-proBNP; turnaround about 10 min for HbA1c, 12 min for NT-proBNP). The AFIAS-3 is an FIA POCT platform with three independent channels, enabling simultaneous testing from a small capillary sample, and providing direct laboratory information system (LIS)/hospital information system (HIS) connectivity features suitable for combined “HbA1c plus NT-proBNP” testing during a single consultation.

Comparators were: 1) HbA1c: Tosoh G8 high-performance liquid chromatography (HPLC) (NGSP-certified, IFCC-traceable); 2) NT-proBNP: cobas 8000 module e602 (Roche Diagnostics), electrochemiluminescence immunoassay (ECLIA). Internal quality control (QC) supplied by the POCT manufacturer was used for precision and bias evaluation. Unless stated otherwise, a single reagent lot was used.

### Reference thresholds

No *de novo* reference intervals were established. Interpretation relied on guideline cut-offs: 1) HbA1c ≥ 6.5% for diagnosis [8, 9]; 2) NT-proBNP ≥ 125 ng/L (ambulatory referral for echocardiography) and ≥ 2,000 ng/L (strongly suggestive of heart failure) [5, 6, 10].

### Participants

Consecutive remnant clinical specimens meeting the required matrices were included: 50 samples per analyte for the method comparison. Samples were deidentified and reflected routine laboratory requests, without enrichment across the analytical range.

#### Inclusion criteria

Adult patients (≥ 18 years) with a physician-ordered HbA1c or NT-proBNP test and a sufficient, properly processed sample.

#### Exclusion criteria

Insufficient volume, clotting, hemolysis, lipemia, mismatched identifiers, or pre-analytical deviations.

### Performance verification

AFIAS-3 POCT performance was assessed for HbA1c and NT-proBNP through QC replicates and 50 patient samples per analyte, compared with Tosoh G8 HPLC and cobas 8000 e602 ECLIA, respectively.

### Precision, accuracy and total error

Internal QC materials were analyzed (five replicates per HbA1c level, 10 replicates per NT-proBNP level). The mean, standard deviation (SD), and coefficient of variation (CV) were calculated. When multi-day repeats were available, the within-laboratory CV was derived; otherwise, within-run repeatability was reported.

Bias versus QC target values was calculated. Observed total error (TE_obs_) was compared to allowable total error (TEa) limits: HbA1c, 6.9% bias calculated in % at 6.5% [8, 9]; NT-proBNP, 30% [11]. Acceptance required TE_obs_ ≤ TEa.

### Method comparison

Fifty patient samples per analyte were tested on AFIAS-3 and the comparator method from the same draw. Samples were included consecutively from routine clinical practice without stratification according to renal function. Passing–Bablok regression (slope, intercept, 95% confidence interval (CI); cumulative sum (CUSUM) test), Bland–Altman analysis (mean bias, limits of agreement), and Spearman’s rank correlation coefficient (ρ) were performed. Matrix agreement was assessed qualitatively when multiple matrices were permissible.

### Diagnostic performance

Classification agreement was evaluated at predefined thresholds (HbA1c 6.5% for diagnosis; NT-proBNP 125 ng/L and 2,000 ng/L). Cohen’s kappa (κ) values were calculated against laboratory comparators.

### User-friendliness of the AFIAS-3

Usability of the POCT device was evaluated with an 11-item questionnaire based on the Scandinavian Evaluation of Laboratory Equipment for Primary Health Care (SKUP) guidelines [12], completed by 10 hospital professionals (five for HbA1c, five for NT-proBNP). Each item was rated as satisfactory, intermediate, or unsatisfactory; overall user-friendliness required a satisfactory rating.

### Statistical analysis

Continuous variables were expressed as mean, SD, and CV. Bias and TE_obs_ were reported for each QC level. Passing–Bablok regression yielded slope and intercept with 95% CI, while Bland–Altman plots illustrated mean bias and ± 1.96 SD limits. Correlation was assessed using Spearman’s ρ, and categorical agreement was quantified by Cohen’s κ. Diagnostic indices were presented with 95% CI, computed in MedCalc v23.2.1 (Ostend, Belgium) using Wilson or exact binomial methods. Statistical analyses were performed with MedCalc and Microsoft Excel (Microsoft 365). A two-sided α = 0.05 was considered statistically significant.

## Results

### Precision and accuracy

The within-run precision of the POCT was acceptable for both HbA1c and NT-proBNP. For HbA1c, the CV was 1.86% at a mean concentration of 5.22% (control 1) and 1.80% at 9.63% (control 2). These results demonstrate very good repeatability, with variability consistently below 2% across both the low and the high measurement ranges (Table 1). The accuracy of HbA1c measurements was assessed against the assigned control values. At the lower level (control 1), the observed bias was 4.50%, corresponding to a total error of 7.6%. This exceeded the 6.9% limit set by the IFCC and NGSP certification criteria. At the higher level (control 2), the bias was 0.80%, yielding an TE_obs_ of 4.3%, which met the most stringent acceptance criteria. Importantly, performance near the diagnostic threshold of 6.5% remained clinically acceptable (Table 1).

**Table 1:** Precision and Accuracy of HbA1c Measurements at Two Quality Control Levels

Analyte	QC level	HbA1c (%)	Mean HbA1c (%)	SD	CV (%)	Bias (%)	TE_obs_ (%)
HbA1c	QC1 (low)	5.47	5.22	0.10	1.86	4.5	7.57
	QC2 (high)	9.71	9.63	0.17	1.80	0.8	4.30

HbA1c: glycated hemoglobin; QC: quality control; SD: standard deviation; CV: coefficient of variation; TE_obs_: total observed error.

For NT-proBNP, the CV was 5.29% at a mean concentration of 249.95 ng/L (control 1) and 15.38% at 5,051.15 ng/L (control 2). Precision was therefore acceptable at the lower clinical decision level but showed greater variability at higher concentrations (Table 2). The bias was 9.11% at the lower level (control 1), corresponding to a TE_obs_ of 17.8%, which was within the 30% limit defined by the Clinical Laboratory Improvement Amendments (CLIA 2025). At the higher level (control 2), the bias was 8.08%, but the TE_obs_ rose to 33.46%, slightly above the allowable 30%. This finding indicates that while performance was acceptable at clinically relevant decision thresholds, the precision of NT-proBNP measurements declined at very high concentrations (Table 2).

**Table 2:** Precision and Accuracy of NT-proBNP Measurements at Two Quality Control Levels

Analyte	QC level	NT-proBNP (ng/L)	Mean NT-proBNP (ng/L)	SD	CV (%)	Bias (%)	TE_obs_ (%)
NT-proBNP	QC1 (low)	275.00	249.95	13.23	5.29	9.11	17.84
	QC2 (high)	5,495.30	5,051.15	776.83	15.38	8.08	33.46

NT-proBNP: N-terminal pro-B-type natriuretic peptide; QC: quality control; SD: standard deviation; CV: coefficient of variation; TE_obs_: total observed error.

### Method comparison

**Figure 1:**
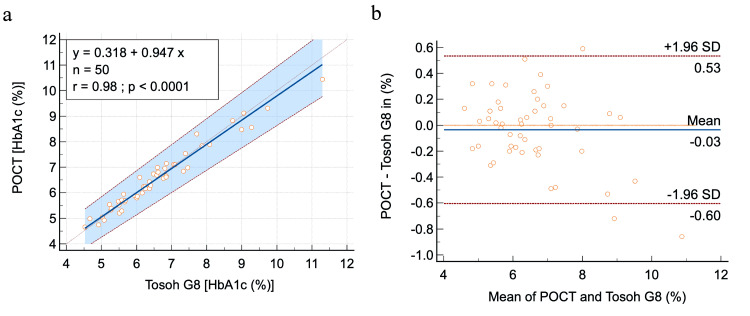
Method comparison between AFIAS-3 POCT and Tosoh G8 HPLC for HbA1c. (a) Passing–Bablok regression showing strong correlation (r = 0.98, P < 0.0001; slope = 0.95; intercept = 0.32%). The blue area indicates the 95% confidence interval. (b) Bland–Altman plot showing a negligible mean bias of −0.03% (blue line) with 95% limits of agreement (red lines) ranging from −0.60% to +0.53%. HbA1c: hemoglobin A1c; POCT: point-of-care testing; HPLC: high-performance liquid chromatography.

For HbA1c, there was a strong correlation between POCT and central laboratory assays (Spearman’s correlation coefficient r = 0.98, P < 0.0001; 95% CI, 0.96–0.99). Passing–Bablok regression yielded an intercept of 0.32% (95% CI, −0.11% to 0.75%) and a slope of 0.95 (95% CI, 0.87–1.02), with no evidence of deviation from linearity (CUSUM test, P = 0.68) (Fig. 1a). Bland–Altman analysis showed a mean bias of −0.03% (95% CI, −0.12% to 0.05%), with limits of agreement from −0.60% to +0.53%. No proportional trend was observed. These results indicate excellent agreement between AFIAS-3 and the reference method (Fig. 1b).

**Figure 2:**
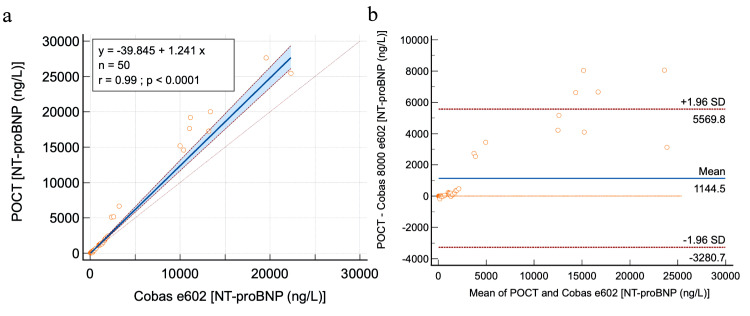
Method comparison between AFIAS-3 POCT and cobas 8000 e602 (ECLIA) for NT-proBNP. (a) Passing–Bablok regression showing very high correlation (r = 0.99, P < 0.0001; slope = 1.24; intercept = −40 ng/L). The blue area indicates the 95% confidence interval. (b) Bland–Altman plot showing a positive mean bias of +1,145 ng/L (blue line) with 95% limits of agreement (red lines) ranging from −3,281 to +5,570 ng/L, reflecting systematic overestimation at higher concentrations. NT-proBNP: N-terminal pro-B-type natriuretic peptide; POCT: point-of-care testing; ECLIA: electrochemiluminescence immunoassay.

For NT-proBNP, the correlation was very high with the central laboratory method (Spearman’s r = 0.99, P < 0.0001; 95% CI, 0.98–1.00). Passing–Bablok regression indicated an intercept of −40 ng/L (95% CI, −64 to −13 ng/L) and a slope of 1.24 (95% CI, 1.17–1.32) (Fig. 2a). In contrast to HbA1c, the CUSUM test showed a significant deviation from linearity (P = 0.03). Bland–Altman analysis revealed a mean positive bias of +1,145 ng/L (95% CI, 503–1,786 ng/L), with wide limits of agreement from −3,281 to +5,570 ng/L. This pattern reflects an increasing overestimation by AFIAS-3 at higher NT-proBNP concentrations, despite the very strong overall correlation with the reference method (Fig. 2b).

### Diagnostic performance

Cohen’s kappa (κ) values indicated consistently strong agreement with the reference methods: κ = 0.92 at the HbA1c 6.5% cut-off, κ = 0.88 at NT-proBNP 125 ng/L, and κ = 0.89 at NT-proBNP 2000 ng/L (Tables 3, 4).

**Table 3:** Categorical Agreement Between AFIAS-3 POCT and Tosoh G8 HPLC for HbA1c at the 6.5% Diagnostic Cut-Off

Cut-off = 6.5%		Reference method Tosoh G8			κ value
		Positive	Negative	Total	
POCT	Positive	26	2	28	0.92
	Negative	0	22	22	
	Total	26	24	50	

HbA1c: glycated hemoglobin; POCT: point-of-care testing; HPLC: high-performance liquid chromatography; κ: Cohen’s kappa.

**Table 4:** Categorical Agreement Between AFIAS-3 POCT and Cobas 8000 e602 for NT-proBNP at Clinical Decision Cut-Offs

	Cobas 8000 module e602			κ value
	Positive	Negative	Total	
Cut-off ≥ 125 ng/L				
POCT				
Positive	10	1	11	0.88
Negative	1	38	39	
Total	11	39	50	
Cut-off ≥ 2,000 ng/L				
POCT				
Positive	37	0	37	0.89
Negative	2	11	13	
Total	39	11	50	

NT-proBNP: N-terminal pro-B-type natriuretic peptide; POCT: point-of-care testing; κ: Cohen’s kappa.

### User-friendliness of the AFIAS-3

Overall, both HbA1c and NT-proBNP assays were rated as user-friendly, with satisfactory scores for sample collection, test execution, result presentation, and functional instructions (Table 5).

**Table 5:** User-Friendliness of the AFIAS-3 for HbA1c and NT-proBNP Point-of-Care Testing

Criteria	Satisfactory (S)	Intermediary (I)	Unsatisfactory (U)	HbA1c results	NT-proBNP results
Device weight	< 5 kg	5–10 kg	> 10 kg	S, S, I, I, U	I, I, I, I, U
Device size	< 5 × 5 cm	5 × 5 cm < x < 30 × 30 cm	> 30 × 30 cm	I, I, I, I, U	I, I, I, I, U
Additional equipment needed (device, cartridges, consumables)	No	1:2	≥ 3	S, S, S, S, I	S, I, I, I, I
Description of the sample collection process	Yes	Incomplete	No	S, S, S, S, S	S, S, I, S, S
Description of the test process	Yes	Incomplete	No	S, S, S, S, S	S, S, S, S, S
Description of how the results are presented	Yes	Incomplete	No	S, S, S, S, S	S, S, S, S, S
Function and use of the test	Yes	Incomplete	No	S, S, S, S, S	S, S, S, S, S
Presence of the necessary instructions for correct use	Yes	Incomplete	No	S, S, S, S, S	S, S, S, I, I
Training required to collect samples	No	Incomplete	Yes	S, S, S, I	S, S, I, I
Training required to use the device	No	Basic training	Special training	S, S, S, S, I	S, S, I, I
Training necessary for calibration and QC	No	Incomplete	Yes	S, S, I, U, U	S, S, S, I, I

HbA1c: glycated hemoglobin; NT-proBNP: N-terminal pro-B-type natriuretic peptide; POCT: point-of-care testing; SKUP: Scandinavian Evaluation of Laboratory Equipment for Primary Health Care; QC: quality control; S: satisfactory; I: intermediary; U: unsatisfactory.

## Discussion

This analytical verification study evaluated the performance of the AFIAS-3 POCT FIA platform for the measurement of HbA1c and NT-proBNP. The results demonstrated excellent analytical agreement with laboratory methods for HbA1c and strong correlation for NT-proBNP.

HbA1c measurements showed excellent precision and minimal bias relative to the reference HPLC method. These results are consistent with previously published evaluations of POCT HbA1c devices [13, 14] and support their potential use in decentralized diagnostic environments [15, 16].

For NT-proBNP, precision was acceptable at lower concentrations but decreased at higher levels, resulting in proportional bias. The observed proportional bias at higher NT-proBNP concentrations warrants careful interpretation, particularly in patients with advanced heart failure or markedly elevated biomarker levels. In such cases, confirmatory testing using central laboratory methods may be advisable. Similar variability has been reported across natriuretic peptide assays [17] and likely reflects differences in antibody specificity and calibration procedures between platforms [18, 19].

Despite this proportional bias, agreement at clinically relevant decision thresholds remained high. This finding suggests that the AFIAS-3 platform may provide reliable results for cardiometabolic biomarker assessment in decentralized care settings [10, 18, 19].

Several limitations should be acknowledged. First, this was a single-center study with a relatively limited sample size, which may affect the generalizability of the findings. In addition, the study focused on analytical performance rather than clinical outcomes. Therefore, larger multicenter studies are warranted to confirm these results and to further assess the clinical and economic impact of such POCT platforms. Furthermore, potential analytical interferences, such as heterophilic antibodies, biotin supplementation, or cross-reactivity with circulating peptide fragments, were not specifically investigated. These factors should be considered when interpreting the results, as previously reported for other immunoassay-based NT-proBNP platforms. Finally, renal function was not systematically documented, although it is likely that patients with varying degrees of renal impairment were included. Given the well-established influence of reduced glomerular filtration rate on NT-proBNP concentrations, this may have contributed to variability in the results and should be more carefully addressed in future studies.

In conclusion, the AFIAS-3 POCT platform demonstrated excellent analytical agreement for HbA1c and acceptable agreement for NT-proBNP at clinically relevant decision thresholds. However, the observed overestimation of NT-proBNP at higher concentrations requires cautious interpretation, and confirmatory testing in central laboratories should be considered in cases of markedly elevated values. These findings support the potential use of this platform for decentralized cardiometabolic biomarker testing. Further studies are needed to confirm clinical utility and cost-effectiveness in real-world settings.

## Data Availability

The data supporting the findings of this study are available from the corresponding author upon reasonable request.
